# Supplementary material for: Sniffer worm, C. elegans, as a toxicity evaluation model organism with sensing and locomotion abilities
Source: PLoS One. 2023 Aug 2;18(8):e0289493. doi: 10.1371/journal.pone.0289493 (PMC10395899; doi:10.1371/journal.pone.0289493)
Supplement: S1 Fig — The control group produced an average of 196 offspring, while C. elegans exposed to 3D-printed (3DP) extracts produced 138 offspring on average. Error bars represent standard error. (PDF) [file pone.0289493.s001.pdf]

Supplemental figure 1

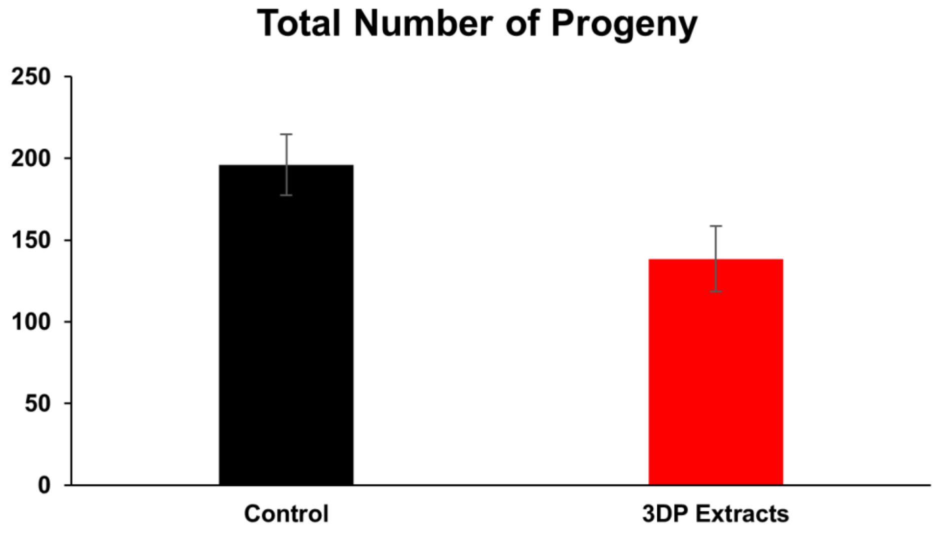

S1 Fig. Total number of progeny. The control group produced an average of 196 offspring, while *C. elegans* exposed to 3D-printed (3DP) extracts produced 138 offspring on average. Error bars represent standard error.
