# Supplementary material for: Sniffer worm, C. elegans, as a toxicity evaluation model organism with sensing and locomotion abilities
Source: PLoS One. 2023 Aug 2;18(8):e0289493. doi: 10.1371/journal.pone.0289493 (PMC10395899; doi:10.1371/journal.pone.0289493)
Supplement: S2 Fig — “5 mM-M” represents 5 mM of 2-methyl-4′-(methylthio)-2-morpholinopropiophenone, and “0.5 mM-M” represents 0.5 mM of it. After L3 stage, “5 mM-M” showed abnormality of development. There were no significant differences observed between control and “0.5 mM-M” groups. Red bars indicate size of 1 mm. (PDF) [file pone.0289493.s002.pdf]

Supplemental figure 2

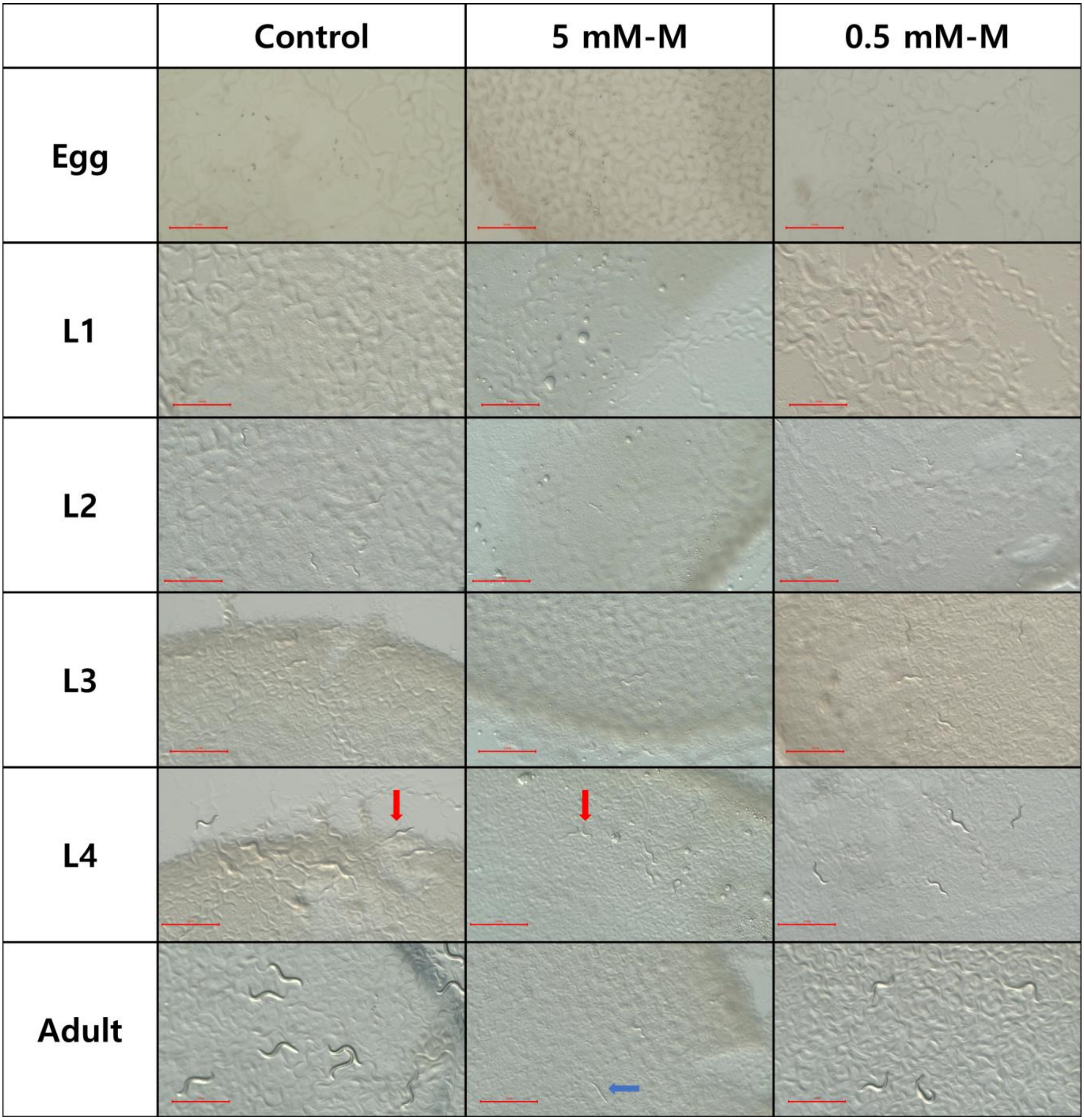

S2 Fig. Effects of photoinitiators on early stage development of *C. elegans*. “5 mM-M” represents 5 mM of 2-methyl-4′-(methylthio)-2-morpholinopropiophenone, and “0.5 mM-M” represents 0.5 mM of it. After L3 stage, “5 mM-M” showed abnormality of development. There were no significant differences observed between control and “0.5 mM-M” groups. Red bars indicate size of 1 mm.
