# Supplementary material for: Sniffer worm, C. elegans, as a toxicity evaluation model organism with sensing and locomotion abilities
Source: PLoS One. 2023 Aug 2;18(8):e0289493. doi: 10.1371/journal.pone.0289493 (PMC10395899; doi:10.1371/journal.pone.0289493)
Supplement: S1 Table — (PDF) [file pone.0289493.s003.pdf]

Supplemental table 1

	Distance from surface of the object	1st	2nd	3rd	Average
Unmasked (cured)	3mm	11	12	29	17.3
	6mm	18	19	63	33.3
	10mm	45	38	59	47.3
	Total	74	69	151	98
Masked (uncured)	3mm	4	5	14	7.7
	6mm	10	19	27	18.7
	10mm	40	46	54	46.7
	Total	54	70	95	73
