# Supplementary material for: Sniffer worm, C. elegans, as a toxicity evaluation model organism with sensing and locomotion abilities
Source: PLoS One. 2023 Aug 2;18(8):e0289493. doi: 10.1371/journal.pone.0289493 (PMC10395899; doi:10.1371/journal.pone.0289493)
Supplement: Attachment — Submitted filename: PONE-D-23-04286_R2_Response to Reviewers.docx [file pone.0289493.s005.docx]

Reviewer #1: The authors have addressed all comments, except for the writing. The revised abstract and introduction is good, figure legends are good, but the 'Results and Discussion' section and "Conclusion" section is mostly left as it was before. In the current state, it is very distracting and difficult to read due to the poor writing, and it is unfit for publication.

The authors must significantly improve the writing to be considered for publication. I will point out a few examples that require attention. I have selected these examples, because these will not be addressed if the authors merely go through some minimal grammar edit, which will not be sufficient.

1. (p. 16, line 376) In the last part of the Results section, there's a part about worm development in 2-methyl-4'-(methylthio)-2-morpholinopropiophenone that was added in the revised manuscript. It's just tacked on in an odd location where it doesn't belong. The section deals with chemotaxis behavior and all of a sudden this data follows, without any transition or explanation. This part should be under its own subheading, maybe after the XPS data where the photoinitiator compound is first mentioned.

<Answer>
Thank you for your guidance. The reviewer’s opinion is correct. We re-located the corresponding paragraph from page 16, line 376-388 to page 14, line 324-339 as you recommended.

- Revised manuscript [Page 14, line 324 - Page 15, line 339]
In fact, XPS analysis showed that the nitrogen 1s signal was detected at 400.2 eV (Fig 3B), indicating that the observed toxicity of the extracted solution might originate from nitrogen-containing photoinitiators. Furthermore, our study on the toxic effects of 2-methyl-4′-(methylthio)-2-morpholinopropiophenone, a widely used photoinitiator in 3D printing resins, on the developmental stage of *C. elegans*. The results showed that abnormal development began at the L3 stage, becoming more pronounced at the L4 stage, with smaller nematode sizes (red arrows) and unhealthy body shape (blue arrow) (S2 Fig). The concentration-dependent toxicity behavior showed that overall behavioral toxicity was barely observed at a 0.5 mM concentration, while toxicity onset occurred at 5 mM. This finding emphasizes the importance of considering local concentration of toxic compounds leaching out from a particular defect point of 3D printed solid materials when assessing toxicity as the effective concentration affecting toxicity in a homogeneously distributed solution may differ. This further highlights the utility of *C. elegans* as a unique model organism for detecting toxic compounds due to its combination of locomotion and sensing abilities.

2. (p. 9, line 195) "Fig 1B clearly shows.." --> the whole sentence structure will have to be fixed, but what I want to point out is, "clearly" is not a good way to describe a result. Whether it is clear or not, the reader will determine. What should be emphasized is whether it is statistically significant, which is a more objective metric.

<Answer>
Thank you for your valuable advice. We have revised the sentence to enhance its objectivity, incorporating your suggestion.

- Original manuscript [Page 9, line 195 - line 197]
Fig 1B clearly shows that the maximal lifespan of *C. elegans* living with the supplement of *E. coli*, which is grown in LB containing toxic compounds extracted from the cuboids, was decreased from 24 (black) to 20 (red) days.

- Revised manuscript [Page 9, line 195 - line 198]
Differences in the maximal lifespan of *C. elegans* were observed in Fig 1B, where the inclusion of *E. coli* supplemented with toxic compounds extracted from the cuboids in LB led to a notable reduction from 24 (black) to 20 (red) days.

3. (p. 11, line 250) "We designed an interesting experiment.." --> again, the readers will determine whether it's interesting or not. Also, these types of fecundity tests are common in the literature, so not sure why you would comment that it's interesting.

<Answer>
Thank you for your valuable advice. We understand your point that the determination of whether an experiment is interesting or not should be left to the readers. Therefore, we have revised the sentence to remove the subjective language.

- Original manuscript [Page 11, line 250 - line 251]
We designed an interesting experiment to observe these toxic effects influencing the fertility of *C. elegans*.

- Revised manuscript [Page 11, line 252 - line 253]
We designed the experiment to observe these toxic effects influencing the fertility of *C. elegans*.

4. (p.13, line 287) "This simple replacement results in a large difference in the toxicity mode of action." --> why is this a large difference? It's different, but is it a "large" difference? The adjective is unnecessary and meaningless, and it inflates the significance of the experiment. If it is large, then it should be followed by more information explaining why it is large.

<Answer>
Thank you for your valuable advice. We have taken your advice and eliminated the unnecessary and exaggerated wording from the sentence, resulting in a more accurate representation of the results.

- Original manuscript [Page 13, line 287 - line 288]
This simple replacement results in a large difference in the toxicity mode of action. The adverse substances are not in the food (i.e., *E. coli*) but rather in water.

- Revised manuscript [Page 13, line 289 - line 290]
The change in the extraction method would result in a difference in the toxicity mode of action.

5. (p.13, line 289) "As C. elegans is able to swim by bending its body in an aqueous solution" --> Because this sentence follows the previous sentence mentioning adverse substance being in water, it sounds like the aqueous solution the worm will swim in is the water extract. The sentence starts with "As", which suggests it's a consequence of something, but when you first read it, it's unclear whether the consequence is what's mentioned before the sentence ("but rather in water") or in the later part of the sentence ("we hypothesized that the speed of..").

<Answer>
Thank you for your valuable advice. We understand your point about the potential confusion caused by the sentence structure and its connection to the preceding statement. To clarify the sequence of events and hypotheses, we have revised the sentence accordingly.

- Original manuscript [Page 13, line 287 - line 294]
This simple replacement results in a large difference in the toxicity mode of action. The adverse substances are not in the food (i.e., *E. coli*) but rather in water. As *C. elegans* is able to swim by bending its body in an aqueous solution, we hypothesized that the speed of bending mobility could be affected. Approximately ten nematodes (age synchronized as 3 days) were transferred to either regular NGM or contaminated NGM by cuboid-extracted water (2 days). Finally, the worms on NGM were washed with M9 buffer and transferred to a 96-well plate to observe the bending speed of each group of *C. elegans*.

- Revised manuscript [Page 13, line 289 - line 299]
The change in the extraction method would result in a difference in the toxicity mode of action. Considering the presence of adverse substances in water not in the food (i.e., *E. coli*), we hypothesized that the speed of *C. elegans*’ bending mobility could be affected. To test this, we selected approximately ten nematodes, all of which were age-synchronized to 3 days. They were then placed in either standard NGM or contaminated NGM by using the cuboid-extracted water, which was prepared for 2 days. *C. elegans* is known to exhibit unique swimming behavior characterized by body bending in an aqueous environment. Therefore, any potential toxicity from the contaminated water might impact this behavior. After the exposure period, the nematodes were removed from the NGM, rinsed with M9 buffer, and transferred to a 96-well plate. This setup allowed us to analyze the bending speeds of the two groups of *C. elegans*.

These are just some examples. I think these reflect the basic expectations of how a research paper should be written, and should be easily fixed if someone more experienced can read over the manuscript.

I am especially troubled by the fact that the extra experiment added in the revised manuscript was carelessly tacked on at the end, in the same paragraph with an unrelated experiment. It is most unprofessional and it confounds me that the authors would go through the trouble of carrying out an extra experiment, but put so little effort in the writing.

<Answer>
Thank you for your comments. The authors went through extensive grammar edits for the rest of paragraphs in results and discussion part as well as conclusion.

**Relative expression levels of a stress-response gene**

- Original manuscript [Page 10, line 226 - Page 11, line 243]
As we observed a lifespan reduction of *C. elegans* exposed to the extract of a 3D-printed cuboid, we assumed that there might be a gene indicator to provide further evidence at the molecular level. We chose hsp-16.2 and sod-3. hsp-16.2 is a stress-responsive reporter gene that predicts longevity in *C. elegans* [22]. The expression level of hsp-16.2 is correlated with increased resistance to heat-shock stress and increased lifespan in *C. elegans* [29]. sod-3 is an antioxidant gene involved in the cellular enzymatic defense system against reactive oxygen species [30]. The worms were exposed to 3D-printed cuboids for 2 days after the adult stage. We observed a significant downregulation of hsp-16.2 in worms treated with 3D-printed cuboid extract (Fig 1C upper panels and 1D left bars). Green fluorescent images were obtained because of the expression of the fusion protein HSP-16.2-GFP (Fig 1C). Qualitative analysis shows that the overall level of green fluorescence was considerably decreased. In addition, quantitative analysis (n = 20 for each group) was also performed. The relative expression level of hsp-16.2 decreased to 52.96 ± 6.37% for the *C. elegans* supplemented with the 3D-printed cuboid extract (Fig 1D, left bars). A similar result was obtained for SOD-3-GFP expression (74.92 ± 6.34% for the 3D cuboid extract group) (Fig 1C lower panels and 1D right bars). Therefore, the nematodes exposed to 3D-printed cuboid extract are thought to be less able to resist stress.

- Revised manuscript [Page 10, line 227 – Page 11, line 245]
As we observed a lifespan reduction of *C. elegans* exposed to the extract of a 3D-printed cuboid, we assumed that there might be a gene indicator to provide further evidences at a molecular level. We selected *hsp-16.2* and *sod-3*. *Hsp-16.2*, a stress-responsive reporter gene, has been shown to predict longevity in *C. elegans*, and its expression correlates with increased resistance to heat-shock stress and enhanced lifespan [22, 29]. *Sod-3*, on the other hand, plays a role in the cellular enzymatic defense system against reactive oxygen species [30]. To investigate these genes, we exposed the worms to 3D-printed cuboids for 2 days post-adulthood. We observed a significant downregulation of hsp-16.2 in worms treated with 3D-printed cuboid extract (Fig 1C upper panels and 1D left bars). The observation was based on green fluorescent images obtained due to the expression of the fusion protein HSP-16.2-GFP (Fig 1C). The qualitative analysis showed a considerable decrease in overall green fluorescence. In addition, quantitative analysis (n = 20 for each group) was also performed. The relative expression level of hsp-16.2 decreased to 52.96 ± 6.37% for the group supplemented with the 3D-printed cuboid extract (Fig 1D, left bars). A comparable result was seen with SOD-3-GFP expression (74.92 ± 6.34% for the 3D cuboid extract group) (Fig 1C lower panels and 1D right bars). These findings suggest that the nematodes exposed to 3D-printed cuboid extract have diminished stress resistance capabilities, reinforcing our previous findings regarding the reduction in lifespan.

***C. elegans* is a unique animal model system with sensing and locomotion abilities**

- Original manuscript [Page 14, line 331 - Page 17, line 388]
As mentioned in the introduction section, *C. elegans* is able to move away from any positions from which harmful toxic compounds are locally present or released. In contrast, its locomotion direction is not affected in nontoxic environments [15]. The chemotactic properties shown by *C. elegans* can point out a source position from which toxic compounds are released. To this point, the experiments described in Figs 1-3 demonstrated that photoinitiators and other toxic compounds were released from the 3D cuboids up to a certain toxicity level. Thus, we designed an interesting experiment when fabricating a 3D cuboid. First, a cuboid with the same dimensions (1 x 1 x 0.1 cm^3^) was printed and then was illuminated with UV light for post-curing to minimize the release of toxic compounds. Second, when the post-curing step was carried out, a piece of Al foil was wrapped around one-half of the side of the cuboid (Fig 4A). We hypothesized that nearly all toxic compounds would be released from the half block without post-UV curing. In fact, the cuboid without post-curing was slightly bent upon applying external force (Fig 4B).

We observed a particular behavior in individual *C. elegans* worms when they closely approached the side of the cuboid without post-curing and then rapidly turned away from the cuboid (< 14 sec) (Fig 4D, upper photos). This 'turn-away' motion could be attributed to formation of a gradient of toxic compounds released from the cuboid. We frequently observed this 'turn-away' motion within a 3 mm distance from the edge of the cuboid. Another observation we made was that some *C. elegans* worms initially crossed over the 3 mm distance while approaching the cuboid, but then turned away from the cuboid (lower photos). In contrast to the turning-away behavior observed in the uncured side, *C. elegans* on the cured control side displayed a different response. The worm approached and moved closer to the cured side without displaying the rapid turning-away motion seen in the uncured side. While occasional turns might still occur on the cured side, they were not as frequent or distinct as the ones observed on the uncured side.

In addition to the aforementioned description of the dynamic motion of an individual *C. elegans*, we observed the overall population behavior in a collection. We observed geometrical distributions of all *C. elegans* organisms centered on the cuboid at 1 hr after worm introduction at the designated four points (Fig 4C). The reason for the 1 hr setting time is to establish the release and gradient formation of toxic substances from the cuboids. We found that *C. elegans* worms were evenly distributed regardless of the Al foil masked or unmasked regions with a distance range from zero to 10 mm (Fig 4E, left bars, S1 Table). In other words, nearly 50% of the population of *C. elegans* was found in the masked region, and the remaining 50% was found in the unmasked region. However, when we looked closely at the region from zero to 6 mm, differences in geometrical distributions were found. Approximately 61% of the *C. elegans* worm were crawling in the photocured region, and 39% of the population was found in the masked region (the middle bars) (p-value = 0.0531). This difference in chemotactic behavior was further enhanced when we observed the crawling worms living from zero to 3 mm from the cuboid. Approximately 70% of the *C. elegans* worms were crawling in the photocured region, and 30% of the population was found in the masked region (the right bars) (p-value < 0.001). The results clearly indicate that *C. elegans* can be a unique model organism for detecting toxic compounds because of the combination of locomotion and sensing abilities. Furthermore, our study on the toxic effects of 2-methyl-4′-(methylthio)-2-morpholinopropiophenone, a widely used photoinitiator in 3D printing resins, on the developmental stage of *C. elegans*. The results showed that abnormal development began at the L3 stage, becoming more pronounced at the L4 stage, with smaller nematode sizes (red arrows) and unhealthy body shape (blue arrow) (S2 Fig). The concentration-dependent toxicity behavior showed that overall behavioral toxicity was barely observed at a 0.5 mM concentration, while toxicity onset occurred at 5 mM. This finding emphasizes the importance of considering local concentration of toxic compounds leaching out from a particular defect point of 3D printed solid materials when assessing toxicity as the effective concentration affecting toxicity in a homogeneously distributed solution may differ. This further highlights the utility of *C. elegans* as a unique model organism for detecting toxic compounds due to its combination of locomotion and sensing abilities.

- Revised manuscript [Page 15, line 351 - Page 17, line 394]
As mentioned in the introduction section, *C. elegans* is able to move away from positions where harmful toxic compounds are locally present or being released, while its locomotion direction remains unaffected in non-toxic environments [15]. The chemotactic properties shown by *C. elegans* can help identify the source of toxic compound release. This is particularly relevant in light of the experiments described in Figs 1-3, which demonstrated that photoinitiators and other toxic compounds were released from the 3D cuboids up to a certain toxicity level. Thus, we designed an experiment when fabricating a 3D cuboid. A cuboid with identical dimensions (1 x 1 x 0.1 cm3) was printed, followed by UV light illumination for post-curing to minimize the release of toxic compounds. Simultaneously, during the post-curing step, we wrapped a piece of Al foil around one half of the cuboid (Fig 4A). Our hypothesis was that majority of toxic compounds would be released from the half block without post-UV curing. In fact, the non-post-cured cuboid exhibited slight bending upon applying external force (Fig 4B).

We observed distinct behavioral patterns in individual *C. elegans* worms when they closely approached the non-post-cured side of the cuboid, rapidly turning away from the cuboid within less than 14 sec. (Fig 4D, upper photos). This 'turn-away' motion may be attributed to the formation of a gradient of toxic compounds released from the cuboid. We frequently observed this 'turn-away' action within a 3 mm distance from the edge of the cuboid. It is noteworthy that some *C. elegans* worms initially crossed over the 3 mm distance while approaching the cuboid, but then quickly turned away from it (lower photos). However, in contrast to the turning-away behavior observed on the non-post-cured side, *C. elegans* approaching the cured side (control) showed a different response. They moved closer to the cured side without displaying the rapid turning-away motion observed on the non-cured side. While occasional turns might still occur on the cured side, they were not as frequent as the ones observed on the uncured side.

Alongside these observations of the individual *C. elegans*, we also examined the overall population behavior. We monitored the geometrical distributions of all *C. elegans* organisms centered on the cuboid, one hour after worm introduction at the designated four points (Fig 4C). We opted for the 1 hr setting time to allow the release and gradient formation of toxic substances from the cuboids. We found that *C. elegans* worms were evenly distributed across both the Al foil masked or unmasked regions within a distance range from zero to 10 mm (Fig 4E, left bars, S1 Table). In other words, approximately 50% of the population of *C. elegans* was found in the masked region, with the remaining 50% located in the unmasked region. Upon further examination of the region from zero to 6 mm, however, we noticed differences in the geometrical distributions. Approximately 61% of the *C. elegans* were found in the photocured region, with 39% located in the masked region (the middle bars) (p-value = 0.0531). This difference in chemotactic behavior became more pronounced when we observed the crawling worms within a range from zero to 3 mm from the cuboid. Approximately 70% of the *C. elegans* worms were located in the photocured region, and 30% in the masked region (the right bars) (p-value < 0.001). The results indicate that *C. elegans* can serve as a unique model organism for detecting toxic compounds because of its combination of locomotion and sensing abilities.

**Conclusion**

- Original manuscript [Page 17, line 404 - Page 18, line 416]
Typical model organisms that have been used in the past have contributed a large amount to toxicity assessments of substances. However, typical model organisms are not suitable for determining in which direction chemicals diffuse from leachable materials. We chose 3D-printed products as typical leachable materials. By using a small nematode, *C. elegans*, approximately 1 mm in body length, we were able to carry out a toxicity test that would be difficult to perform with conventional model organisms. The 3D-printed block extract adversely affected the health of *C. elegans*, resulting in reduced lifespan, reduced exercise capacity, reduced number of offspring, and decreased relative expression levels of stress-response genes. In addition, the leaching point and the direction of chemical diffusion could be monitored by following the movement of worms located near the substances. Material toxicity assessments that could not have been conducted with conventional model organisms can be performed by tracking the distribution and behavior of *C. elegans*.

- Revised manuscript [Page 18, line 410 - line 426]
Conventional model organisms that have greatly contributed to toxicity assessments of substances in the past. However, these typical model organisms do not offer an adequate solution for discerning the direction in which chemicals diffuse from leachable materials. In this study, we selected 3D-printed products as representative leachable materials and used a small nematode, *C. elegans*, which is approximately 1 mm in body length, for our toxicity test. This approach allowed us to conduct a toxicity test that would be challenging to perform with traditional model organisms. Our results showed that the extract from the 3D-printed block adversely affected the health of *C. elegans*, leading to a shortened lifespan, reduced physical activity, diminished offspring count, and decreased relative expression levels of stress-response genes. Furthermore, by tracking the movement of the worms in proximity to the substance, we were able to monitor the leaching point and the direction of chemical diffusion of toxic components. This research demonstrates that material toxicity assessments, which could not have been conducted with conventional model organisms, can now be performed by monitoring the distribution and behavior of *C. elegans*. As such, *C. elegans* proves itself to be a valuable model organism, offering insight into the understanding and assessment of material toxicity in a dynamic manner.
