# Supplementary material for: Sniffer worm, C. elegans, as a toxicity evaluation model organism with sensing and locomotion abilities
Source: PLoS One. 2023 Aug 2;18(8):e0289493. doi: 10.1371/journal.pone.0289493 (PMC10395899; doi:10.1371/journal.pone.0289493)
Supplement: Attachment — Submitted filename: PONE-D-23-04286_R3_Response to Reviewers.docx [file pone.0289493.s006.docx]

6. Review Comments to the Author

Reviewer #1: Despite the two rounds of revisions, the writing is still very poor. The edits made are insufficient to bring it up to the level fit for publication.

Thank you very much for your comments.

The authors carried out comprehensive editing and revision throughout the manuscript keeping efforts not to change the original meaning of each sentence.

**Abstract**

Three-dimensional (3D) printed objects have a higher likelihood of containing local defects compared to products manufactured by conventional casting-based methods.

🡪 Additive manufacturing, or 3D printing, has revolutionized the way we create objects. However, its layer-by-layer process may lead to an increased incidence of local defects compared to traditional casting-based methods.

The multistep, layer-by-layer process in additive manufacturing, with factors such as light intensity, variations of light penetration depth, inhomogeneity of components, and fluctuation of nozzle temperature, contribute to the increased defect generations.

🡪 Factors such as light intensity, depth of light penetration, component inhomogeneity, and fluctuations in nozzle temperature all contribute to defect formations.

Defect regions are sources of toxic component release, but methods to identify them in printed materials have not been reported.

🡪 These defective regions can become sources of toxic component leakage, but pinpointing their locations in 3D printed materials remains a challenge.

Traditional toxicological assays rely on extraction followed by treatments to living creatures, which is a passive detection of harmful agents.

🡪 Traditional toxicological assessments rely on the extraction and subsequent exposure of living organisms to these harmful agents, thus only offering a passive detection approach.

Thus, developing of an active system to identify and locate the sources of toxicity is a critical issue in 3D printing technologies.

🡪 Therefore, the development of an active system to both identify and locate sources of toxicity is essential in the realm of 3D printing technologies.

Herein, we present the use of the nematode model system, *C. elegans*, for toxicity evaluation.

🡪 Herein, we introduce the use of the nematode model organism, *Caenorhabditis elegans* (*C. elegans*), for toxicity evaluation.

*C. elegans* displays unique ‘sensing’ and ‘locomotion’ abilities, actively moving toward safe regions while avoiding toxic areas.

🡪 *C. elegans* exhibits distinctive 'sensing' and 'locomotion' capabilities that enable it to actively navigate toward safe zones while steering clear of hazardous areas.

These capabilities make *C. elegans* an unparalleled choice among existing aquatic and animal models, offering immediate and precise indications to locate and identify the sources of toxicity in 3D printed materials.

🡪 This active behavior sets *C. elegans* apart from other aquatic and animal models, making it an exceptional choice for immediate and precise identification and localization of toxicity sources in 3D printed materials.

**Introduction**

The assessment necessitates the use of living organisms to determine material toxicity

🡪 Assessment of material toxicity fundamentally requires testing on living organisms.

This plant’s small size, easy of cultivation, and short life cycle make it a practical choice for toxicity evaluations [3,4]

🡪 *Lemna gibba*'s small size, simple cultivation process, and short life cycle render it a practical candidate for toxicity assessments [3,4]

Primary advantage of using this plant lies in the ease of observing growth inhibition by toxic components.

🡪 The primary advantage of utilizing this plant model resides in the observation of growth inhibition induced by toxic elements.

However, the results obtained from *Lemna gibba* growth inhibition did not directly correspond to toxicological effects on humans.

🡪 However, the outcomes obtained from *Lemna gibba* growth inhibition studies do not directly parallel the toxicological impacts on humans.

Due to the limitations associated with the correlation between plant-based toxicity models and human toxicity levels, later studies have introduced animal models.

🡪 Due to the discrepancies between plant-based toxicity models and human toxicity outcomes, subsequent research has embraced the use of animal models.

However, the results obtained in aquatic environments are very different from those in ambient conditions, leading to a preference for terrestrial animal models.

🡪 However, the findings from aquatic settings vary significantly from those observed in terrestrial conditions, thus inclining researchers towards the use of terrestrial animal models.

However, implementation of stringent and wide-ranging ethical regulations has recently posed a significant challenge to the use of these model systems [10].

🡪 Nonetheless, the recent enforcement of rigorous and comprehensive ethical regulations has introduced considerable challenges to the employment of these model systems [10].

Reasons include that layer-by-layer deposition processes in additive manufacturing essentially involves numerous repeated steps, during which unavoidable defects are generated.

🡪 This is primarily due to the intrinsic layer-by-layer deposition processes in additive manufacturing, which inherently involve multiple iterations, during which the generation of defects is inescapable.

In stereolithography (SLA) methods, variations in light intensity, penetration depth, and inhomogeneity of polymers concentrations and dispersion can occur [13].

🡪 In stereolithography (SLA) methods, variances in factors such as light intensity, penetration depth, and the concentration and dispersion inhomogeneity of polymers can arise [13].

Furthermore, the irradiation with UV-light leads to a homolytic bondage cleavage and the generation of highly reactive radical species, which are the origin of chemical toxicity.

🡪 Furthermore, UV-light irradiation triggers a homolytic bond cleavage, resulting in the creation of highly reactive radical species, which serve as the roots of chemical toxicity.

In fused deposition modeling (FDM), nozzle temperature fluctuations contribute to the formation of local defects during printing [14].

🡪 In fused deposition modeling (FDM), fluctuations in nozzle temperature significantly contribute to the development of local defects during the printing process [14].

As 3D printing techniques are ideal for small-scale prototype production, each product may display unique sites of defects, resulting in varying levels of toxicity.

🡪 Given that 3D printing techniques are optimally suited for small-scale prototype production, each product may exhibit distinctive defect sites, thereby leading to disparate levels of toxicity.

Thus, there is a need to develop a new animal model capable of detecting product-to-product variations in toxicity sources.

🡪 Consequently, it becomes imperative to develop a novel animal model capable of detecting variations in toxicity sources between individual products.

Plant models lack the locomotive function to identify toxicological sources within printed materials.

🡪 Plant models, lacking locomotive capabilities, fail to discern toxicological sources within printed materials.

While zebrafish, an aquatic toxicity evaluation model, has a locomotive function, leached toxic compounds become diffused in water, making them unsuitable for pinpointing toxicity sources in printed materials.

🡪 Although zebrafish, an aquatic model used in toxicity evaluations, possess locomotive abilities, the diffusion of leached toxic compounds in water impedes their effectiveness in accurately locating toxicity sources within printed materials.

In light of these challenges, a new toxicity model should combine both sensing and locomotion capabilities.

🡪 Given these challenges, it becomes critical to introduce a toxicity model that unifies both sensing and locomotion capabilities.

Here, we propose the use of C. elegans as a model capable of detecting both the overall toxicity and specific toxic sites within printed materials.

🡪 We propose the employment of *Caenorhabditis elegans* (*C. elegans*), a model endowed with the capacity to discern both the overall toxicity and specific toxic sites within printed materials.

The nematode’s crawling activity serves as phenotypic indicators for identifying the sources of toxicity within test materials.

🡪 The crawling activity of the nematode serves as phenotypic indicators pivotal for pinpointing the sources of toxicity within test materials.

However, the potential of C. elegans as an ‘active’ toxicological model, incorporating both sensing and locomotion abilities, has not been explored previously.

🡪 Yet, the potential of C. elegans as an 'active' toxicological model, integrating both sensing and locomotion capabilities, has been unexplored.

**Results and Discussion**

**- Original manuscript [Page 8, line 189 – Page 9, line 201]
Effect of 3D printed blocks extracted LB on lifespan**

We hypothesized the following. First, Luria-Bertani broth (LB) media were prepared because the energy source of *C. elegans* is bacteria, for which *Escherichia coli* (OP50) has been widely used. Second, any toxic compounds released from 3D-printed cuboids (1 x 1 x 0.1 cm^3^, 80 units incubation in 400 mL of LB, Fig 1A) might be adsorbed during *E. coli* growth. Third, the *C. elegans* eating the contaminated *E. coli* might exhibit a reduced lifespan. Differences in the maximal lifespan of *C. elegans* were observed in Fig 1B, where the inclusion of *E. coli* supplemented with toxic compounds extracted from the cuboids in LB led to a notable reduction from 24 (black) to 20 (red) days. A control experiment used LB media without toxic compound extraction from the cuboids. Importantly, the mean lifespan, defined by 50% survival of *C. elegans*, was 15 days for the control, which was decreased to approximately 13 days for the treatment group (dotted lines, Fig 1B).

**- Revised manuscript [Page 9, line 194 – line 205]
Toxic substance effects on *C. elegans*’ lifespan**

Release of toxic compounds released from the 3D-printed cuboids was monitored by observing the lifespan of *C.elegans*. Fig 1B shows the differences in the maximal lifespan of *C. elegans*. When *C.elegans* was exposed to the toxic compounds through the *Escherichia. Coli* (OP50) supplemented with toxic compounds extracted from the cuboids in Luria-Bertani broth (LB) media, the lifespan was reduced from 24 (black) to 20 (red) days. We hypothesized that toxic compounds released from the 3D-printed cuboids are absorbed by OP50 during the growth in LB media. A control experiment was carried out with the OP50 grown in LB media without the toxic compounds extracted from the cuboids. The mean lifespan, defined by 50% survival of *C. elegans*, was 15 days for the control, which was decreased to 13 days for the treatment group (dotted lines, Fig 1B).

**- Original manuscript [Page 10, line 227 - line 245]**
As we observed a lifespan reduction of *C. elegans* exposed to the extract of a 3D-printed cuboid, we assumed that there might be a gene indicator to provide further evidences at a molecular level. We selected *hsp-16.2* and *sod-3*. *Hsp-16.2*, a stress-responsive reporter gene, has been shown to predict longevity in *C. elegans*, and its expression correlates with increased resistance to heat-shock stress and enhanced lifespan [22, 29]. *Sod-3*, on the other hand, plays a role in the cellular enzymatic defense system against reactive oxygen species [30]. To investigate these genes, we exposed the worms to 3D-printed cuboids for 2 days post-adulthood. We observed a significant downregulation of *hsp-16.2* in worms treated with 3D-printed cuboid extract (Fig 1C upper panels and 1D left bars). The observation was based on green fluorescent images obtained due to the expression of the fusion protein HSP-16.2-GFP (Fig 1C). The qualitative analysis showed a considerable decrease in overall green fluorescence. In addition, quantitative analysis (n = 20 for each group) was also performed. The relative expression level of hsp-16.2 decreased to 52.96 ± 6.37% for the group supplemented with the 3D-printed cuboid extract (Fig 1D, left bars). A comparable result was seen with SOD-3-GFP expression (74.92 ± 6.34% for the 3D cuboid extract group) (Fig 1C lower panels and 1D right bars). These findings suggest that the nematodes exposed to 3D-printed cuboid extract have diminished stress resistance capabilities, reinforcing our previous findings regarding the reduction in lifespan.

**- Revised manuscript [Page 10, line 230 – Page 11, line 248]**
The lifespan reduction of *C. elegans* exposed to the extract of a 3D-printed cuboid indicates that there might be a gene indicator to provide further evidences at a molecular level. We selected *hsp-16.2* and *sod-3*. *Hsp-16.2*, a stress-responsive reporter gene, has been known to predict longevity in *C. elegans*, and its expression correlates with increased resistance to heat-shock stress and enhanced lifespan [22, 29]. *Sod-3*, on the other hand, plays a role in the cellular enzymatic defense system against reactive oxygen species [30]. When the worms were exposed to the 3D-printed cuboid extracts for 2 days post-adulthood, we observed a significant downregulation of *hsp-16.2* in the worms treated with 3D-printed cuboid extract (Fig 1C upper panels and 1D left bars). The observation was made with the green fluorescent images due to the expression of the fusion protein HSP-16.2-GFP (Fig 1C). The qualitative analysis showed a considerable decrease in overall green fluorescence. In addition, quantitative analysis (n = 20 for each group) was also performed. The relative expression level of hsp-16.2 decreased to 52.96 ± 6.37% for the group supplemented with the 3D-printed cuboid extracts (Fig 1D, left bars). A comparable result was obtained with SOD-3-GFP expression (74.92 ± 6.34% for the 3D cuboid extract group) (Fig 1C lower panels and 1D right bars). These findings suggest that the nematodes exposed to 3D-printed cuboid extracts have diminished the stress resistance capabilities, supporting our previous findings regarding the reduction in lifespan.

**- Original manuscript [Page 11, line 247 - line 253]**
We assume that the toxic substances released from the cuboids might affect the fertility of *C. elegans*. In general, approximately 300 eggs are hatched in the entire lifetime of a healthy nematode [31]. Thus, tens of eggs can be found in a plate even after one day of culture of a single *C. elegans* organism [32]. Negative effects on fertility can be observed for *C. elegans* organisms that are exposed to the toxic substances released into LB. We designed the experiment to observe these toxic effects influencing the fertility of *C. elegans*. First, all *C. elegans* nematodes were synchronized in their age.

**- Revised manuscript [Page 11, line 245 – line 251]**
We thought that the toxic substances released from the cuboids could impact the fertility of *C. elegans*. Normally, healthy nematode can hatch approximately 300 eggs throughout its lifetime [31], resulting in the presence of tens of eggs even after one day of culturing a single *C. elegans* organism [32]. Exposure to the toxic substances in LB medium can adversely affect the fertility of *C. elegans*. Thus, we designed an experiment to investigate the influence of these toxic effects on their fertility. Firstly, all *C. elegans* nematodes were synchronized in their age.

**- Original manuscript [Page 12, line 285 – Page 13, line 309]**
For the previous LB case, we observed overall negative toxicity results regarding lifespan, stress-response genes, and fertility. These results are due to the contaminated *E. coli* bacteria that were prepared in media into which toxic substances had been released. A different type of extraction is replacing LB media with distilled water (Fig 2C). The change in the extraction method would result in a difference in the toxicity mode of action. Considering the presence of adverse substances in water not in the food (i.e., *E. coli*), we hypothesized that the speed of *C. elegans’* bending mobility could be affected. To test this, we selected approximately ten nematodes, all of which were age-synchronized to 3 days. They were then placed in either standard NGM or contaminated NGM by using the cuboid-extracted water, which was prepared for 2 days. *C. elegans* is known to exhibit unique swimming behavior characterized by body bending in an aqueous environment. Therefore, any potential toxicity from the contaminated water might impact this behavior. After the exposure period, the nematodes were removed from the NGM, rinsed with M9 buffer, and transferred to a 96-well plate. This setup allowed us to analyze the bending speeds of the two groups of *C. elegans*. We counted the number of body bends for 1 minute. Fig 2E shows time-lapse photo arrays representing body bending. For a healthy *C. elegans* worm, it takes approximately 1.6 sec to bend its body to an opposite position (upper photos). However, we observed that twice the time was required for the same bending motion in the case of the exposed worms (lower photos). Quantitative analysis showed that the unexposed worms (control) bent 53.2 times per minute (n = 6), which was decreased to 24 bends per minute for the exposed *C. elegans* (n = 6) (Fig 2D). Although we cannot exclude the possibility of the OP50 taking up the toxins from the NGM media, these results clearly demonstrate the adverse effect of contaminated water produced from cuboid extraction on *C. elegans* motility.

**- Revised manuscript [Page 13, line 286 – Page 14, line 310]**
The overall negative effects on lifespan, stress-response genes, and fertility were attributed to the toxic substances released into the media which contaminated *E. coli* bacteria. To investigate a different mode of toxicity effect, we employed a different extraction method by replacing LB media with distilled water (Fig 2C). This change in the extraction method provides a difference in the toxicity mode of action. The presence of toxic substances in water rather than in the food (i.e., *E. coli*) may induce the change of *C. elegans’* bending mobility. To test this, we selected approximately ten nematodes, all of which were age-synchronized to 3 days. They were then placed in either standard NGM or contaminated NGM by using the cuboid-extracted water. *C. elegans* is known to exhibit unique swimming behavior characterized by body bending in an aqueous environment. Therefore, any potential toxicity in the contaminated water might affects this behavior. After the exposure, the nematodes were removed from the NGM, rinsed with M9 buffer, and transferred to a 96-well plate. This setup allowed us to analyze the bending speeds of the two groups of *C. elegans*. We counted the number of body bends for 1 minute. Fig 2E shows time-lapse photo arrays representing body bending. For a healthy *C. elegans* worm, it takes approximately 1.6 sec to bend its body to an opposite position (upper photos). However, we observed that twice the time was required for the same bending motion in the case of the exposed worms (lower photos). Quantitative analysis showed that the unexposed worms (control) bent 53.2 times per minute (n = 6), which was decreased to 24 bends per minute for the exposed *C. elegans* (n = 6) (Fig 2D). Although we cannot exclude the possibility of the OP50 taking up the toxins from the NGM media, these results show the adverse effect of contaminated water with the cuboid extracts on *C. elegans* motility.

**- Original manuscript [Page 14, line 313 - line 329]**
In general, biological toxicity when using photocurable resins is largely dependent on the photoinitiators [33]. However, knowledge of the resin composition used in this study is quite limited due to the company’s proprietary right. Nevertheless, it is expected that the resin contains at least a certain amount of photoinitiators. We thought that unreacted photoinitiators would remain inside of the 3D-printed products until they eventually leached out and adversely affected the health of *C. elegans*. In Fig 3A, the chemical structures of widely used UV-curable photoinitiators are presented: 2-benzyl-2-(dimethylamino)-4’-morpholinobutyrophenone, 4-(dimethylamino)benzophenone, azobisisobutyronitrile, and 2-methyl-4-2-morpholinopropiophenone. Nitrogen atoms commonly exist because they undergo homolytic cleavage upon introducing light energy [34]. For this reason, we assumed that the extracted solution might have nitrogen-containing compounds. In fact, XPS analysis showed that the nitrogen 1s signal was detected at 400.2 eV (Fig 3B), indicating that the observed toxicity of the extracted solution might originate from nitrogen-containing photoinitiators. Furthermore, our study on the toxic effects of 2-methyl-4′-(methylthio)-2-morpholinopropiophenone, a widely used photoinitiator in 3D printing resins, on the developmental stage of *C. elegans*.

**- Revised manuscript [Page 14, line 314 – line 329]**
Biological toxicity of the photocurable resins comes from the photoinitiators [33]. However, the detailed information of the resin composition used in this study is quite limited due to the company’s proprietary right. Nevertheless, we thought that the resin contains certain amount of photoinitiators. We hypothesized that unreacted photoinitiators would remain within the 3D-printed products until they leach out over time, potentially causing adverse effect on the health of *C. elegans*. Fig 3A illustrates the chemical structures of widely used UV-curable photoinitiators: 2-benzyl-2-(dimethylamino)-4’-morpholinobutyrophenone, 4-(dimethylamino)benzophenone, azobisisobutyronitrile, and 2-methyl-4-2-morpholinopropiophenone. These compounds have nitrogen atoms, which undergo homolytic cleavage upon exposure to light energy [34]. Consequently, the extracted solution contains the nitrogen-containing compounds. In fact, XPS analysis revealed that the nitrogen 1s signal was detected at 400.2 eV (Fig 3B), indicating that the observed toxicity of the extracted solution might originate from the nitrogen-containing photoinitiators. Further, we investigated the toxic effects of 2-methyl-4′-(methylthio)-2-morpholinopropiophenone, a widely used photoinitiator in 3D printing resins, on the developmental stage of *C. elegans*.

**- Original manuscript [Page 15, line 351 – Page 16, line 364]**
As mentioned in the introduction section, *C. elegans* is able to move away from positions where harmful toxic compounds are locally present or being released, while its locomotion direction remains unaffected in non-toxic environments [15]. The chemotactic properties shown by *C. elegans* can help identify the source of toxic compound release. This is particularly relevant in light of the experiments described in Figs 1-3, which demonstrated that photoinitiators and other toxic compounds were released from the 3D cuboids up to a certain toxicity level. Thus, we designed an experiment when fabricating a 3D cuboid. A cuboid with identical dimensions (1 x 1 x 0.1 cm^3^) was printed, followed by UV light illumination for post-curing to minimize the release of toxic compounds. Simultaneously, during the post-curing step, we wrapped a piece of Al foil around one half of the cuboid (Fig 4A). Our hypothesis was that majority of toxic compounds would be released from the half block without post-UV curing. In fact, the non-post-cured cuboid exhibited slight bending upon applying external force (Fig 4B).

**- Revised manuscript [Page 15, line 351 – Page 16, line 364]**
As mentioned in the introduction section, *C. elegans* possesses the ability to detect and move away from locations where harmful toxic compounds are present or being released, while maintaining its locomotion in non-toxic environments [15]. The chemotactic behavior displayed by *C. elegans* can be leveraged to identify the source of toxic compound release. In the context of the experiments described in Figs 1-3, which demonstrated that photoinitiators and other toxic compounds could be released from the 3D cuboids up to a certain toxicity level. Thus, we designed an experiment when fabricating a 3D cuboid. A cuboid with identical dimensions (1 x 1 x 0.1 cm^3^) was printed, followed by UV light illumination for post-curing to minimize the release of toxic compounds. Simultaneously, during the post-curing step, we wrapped a piece of Al foil around one half of the cuboid (Fig 4A). Our hypothesis was that majority of toxic compounds would be released from the half block without post-UV curing. Indeed, the non-post-cured cuboid exhibited slight bending upon applying external force (Fig 4B).

**- Original manuscript [Page 18, line 410 - line 426]**
Conventional model organisms that have greatly contributed to toxicity assessments of substances in the past. However, these typical model organisms do not offer an adequate solution for discerning the direction in which chemicals diffuse from leachable materials. In this study, we selected 3D-printed products as representative leachable materials and used a small nematode, *C. elegans*, which is approximately 1 mm in body length, for our toxicity test. This approach allowed us to conduct a toxicity test that would be challenging to perform with traditional model organisms. Our results showed that the extract from the 3D-printed block adversely affected the health of *C. elegans*, leading to a shortened lifespan, reduced physical activity, diminished offspring count, and decreased relative expression levels of stress-response genes. Furthermore, by tracking the movement of the worms in proximity to the substance, we were able to monitor the leaching point and the direction of chemical diffusion of toxic components. This research demonstrates that material toxicity assessments, which could not have been conducted with conventional model organisms, can now be performed by monitoring the distribution and behavior of *C. elegans*. As such, *C. elegans* proves itself to be a valuable model organism, offering insight into the understanding and assessment of material toxicity in a dynamic manner.

**- Revised manuscript [Page 18, line 410 – line 426]**
Conventional model organisms have played a significant role in past toxicity assessments of various substances. However, these typical models lack the capability to accurately determine the direction of chemical diffusion from leachable materials. In this study, we employed 3D-printed products as representative leachable materials and utilized the small nematode *C. elegans*, which measures approximately 1 mm in body length, for our toxicity test. This approach enabled us to conduct a toxicity assessment that would have been challenging with traditional model organisms. Our findings showed that the extract obtained from the 3D-printed block had detrimental effects on the health of *C. elegans*, resulting in a shortened lifespan, reduced physical activity, decreased offspring count, and diminished expression levels of stress-response genes. Moreover, by tracking the movement of the worms in the vicinity of the substance, we were able to observe the leaching point and ascertain the direction of chemical diffusion of the toxic components. This research demonstrates the utility of *C. elegans* as a model organism, providing insights into the understanding and evaluation of material toxicity in a dynamic manner. Consequently, material toxicity assessments that were previously impractical with conventional model organisms can now be performed by monitoring the distribution and behavior of *C. elegans*.
